# Developing a patient and family-centred approach for measuring the quality of injury care: a study protocol

**DOI:** 10.1186/1472-6963-13-31

**Published:** 2013-01-27

**Authors:** Henry T Stelfox, Jamie M Boyd, Sharon E Straus, Anna R Gagliardi

**Affiliations:** 1 Department of Critical Care Medicine, University of Calgary and Alberta Health Services, Calgary Zone, Canada; 2 Department of Medicine, University of Calgary and Alberta Health Services, Calgary Zone, Canada; 3 Department of Community Health Sciences, University of Calgary, Calgary, Canada; 4 Institute for Public Health, University of Calgary, Calgary, Canada; 5 Saint Michael’s Hospital, Department of Medicine, University of Toronto, Toronto, ON, Canada; 6 University Health Network, Toronto, ON, Canada; 7 Teaching Research & Wellness Building, University of Calgary, 3280 Hospital Drive NW, Calgary, AB, T2N 4Z6, Canada

**Keywords:** Injury, Patient-centred care, Quality improvement, Quality indicators, Trauma, Focus group, Consensus panel, Survey

## Abstract

**Background:**

Quality indicators (QI) are used in health care to measure quality of service and performance improvement. Health care professionals and organizations caring for patients with injuries need information regarding the quality of care provided and the outcomes experienced in order to target improvement efforts. However, very little is known about the quality of injury care provided to individual patients and populations and even less about patients’ perspectives on quality of care. The absence of QIs that incorporate patient or family preferences, needs or values has been identified as an important gap in the science and practice of injury quality improvement. The primary objective of this research protocol is to develop and evaluate the first set of patient and family-centred QIs of injury care for critically injured patients

**Methods/design:**

This mixed methods study is comprised of three Sub-Studies. *Sub-Study A* will utilize focus group methodology to describe the preferences, needs and values of critically injured patients and their family members regarding the quality of health care delivered. Qualitative content analysis of the transcripts will begin after the first completed focus group and will draw on grounded theory using a process of open, axial and selective coding. A panel of stakeholders will be assembled during *Sub-Study B* to review the themes identified from the focus groups and develop a catalogue of potential patient and family-centred QIs of injury care using the RAND/UCLA Appropriateness Method (RAM). The QIs developed by the stakeholder panel will be pilot tested in *Sub-Study C* using surveys of patients and their family members to determine construct validity, intra-rater reliability and clinical sensibility.

**Discussion:**

Measuring the quality of injury care is but a first step towards improving patient outcomes. This research will develop the first set of patient and family-centred QIs of injury care. To improve patient care, we need accessible, reliable indicators of quality that are important to patients, and that can then be used to establish quality of care benchmarks, to flag potential problems or successes, follow trends over time and identify disparities across organizations, communities, populations and regions.

## Background

Each year, injuries affect 700 million people worldwide [1,2] with more than five million people dying from injuries annually [3]. The human and societal burden of injuries is even greater with many survivors being permanently impaired and never returning to school, work or their “regular” lives [4,5]. Evaluations of the quality of care in medicine have revealed that medical care often falls short of established standards, and this is also true of injury management. Half of all critically injured patients do not receive recommended care [6-10], medical errors are common in this population [11,12] and preventable injury deaths in hospital are widely reported [13-17].

These studies were based on evaluation of guideline recommendations, but injury management is complex. Performance measures reflecting a range of decisions, processes and outcomes are needed to truly measure care delivery and its impact, and identify whether and how improvements are needed. To address the challenge of providing valid and reliable measurement of injury care quality, we implemented a research program to develop population-based and evidence-based quality indicators (QI) of injury care. A *Research Synthesis* of existing QIs of injury care in published and unpublished literature [18,19] identified a large heterogeneous group of indicators (n = 1,572 QIs from 192 articles in three languages) supported by a limited evidence base [18-20]. Based on the results of the S*ynthesis*, an *International Audit of QI Practices* was performed by conducting surveys and interviews of the leaders of 251 accredited North American and Australasian trauma centres. Ninety seven percent of participating centres employ QIs to measure the quality of injury care they deliver. However, this work identified an important gap in the science and practice of injury quality improvement, the absence of QIs that incorporate patient or family preferences, needs or values (n = 0/1,572 QIs identified in *Research Synthesis* and n = 19/11,460 QIs identified in *International Audit of QI Practices*).

The Institute of Medicine (IOM) defines quality as “the degree to which health care services for individuals and populations increase the likelihood of desired outcomes and are consistent with current professional knowledge.”[21] Central to this definition is that desired outcomes be consistent with both clinical goals and the patients’ goals. In *Crossing the Quality Chasm*, the IOM emphasized the importance that care be *patient-centred*, “respectful of and responsive to individual patient preferences, needs, and values.”[22] At present, patient preferences are best understood for primary care and chronic diseases [23]. Conversely, relatively little is known about the priorities of critically injured patients who epitomize the challenges of providing patient-centred acute care. Surveys of traumatic brain injured patients and their family members have demonstrated that many perceive a significant information deficit from health professionals [24,25]. Janssen et al. [26] demonstrated satisfaction with hospital care of patients with injuries is associated with perceptions of being involved in treatment decisions, being attended to by physicians and trusting physicians. While these are important initial learnings more information is needed.

As healthcare systems seek to provide patient-centred care, questions about how to measure this aspect of quality have become more important since previously developed QIs rarely incorporated patient perspectives [19,27]. A qualitative study done in preparation for this research demonstrated patients of differing age, sex and health status have consistently indicated that patient participation is important so that quality improvement efforts can reflect patient preferences, something that cannot be determined by health providers [28,29]. Patient-centred measures have been successfully developed in select domains of health care. For example, the Consumer Assessment of Health Providers and Systems Survey (CAHPS) has been used to evaluate patient experiences with primary care, and has identified shortfalls in care delivery and support quality improvement [30-32]. A hospital version of CAHPS (HCAHPS) has been developed to measure medical, surgical and obstetrical inpatient experiences with care, is publicly reported in the United States [33] and the results appear to correlate with processes of care for acute myocardial infarction, congestive heart failure, pneumonia and prevention of complications from surgery [34]. No such patient-centred QIs currently exist to evaluate the quality of injury care.

### Aim

The primary aim of this study is to develop and evaluate the first set of patient and family-centred quality indicators of injury care for critically injured patients. These indicators will be designed to reflect the emerging health needs of critically injured patients and to support health policy decision making to improve the quality of injury care [35]. We define QIs as performance measures that compare actual care against ideal criteria [22,36]. The QIs will be patient and family-centred, reflecting the preferences, needs and values of patients and their family members [22]. We define critically injured patients as those with injuries from “the physical damage that results when a human body is suddenly subjected to energy in amounts that exceed the threshold of physiological tolerance” [37] resulting in admission to an intensive care unit, a step-down unit or a monitored, high acuity unit. The specific objectives of the proposed study are:

• To determine the preferences, needs and values of recovering critically injured patients and family members from four trauma centres in Canada regarding quality injury care (*Sub-Study A*) including: the most important dimensions of quality of injury care; how quality of injury care should be measured; how quality should be reported; and how quality should be improved.

• To develop patient and family-centred QIs in injury care using a multi-step development process (*Sub-Study B*).

• To pilot test the patient and family-centred QIs for construct validity, intra-rater reliability, and clinical sensibility (*Sub-Study C*).

## Methods/design

### Approach

This mixed methods study is comprised of three S*ub-Studies*: A) Patient & Family Focus Groups, B) Multi-Step Quality Indicator Development Process and C) Pilot Test of Quality Indicators. The qualitative components of this research adhere to the RATS guidelines for qualitative research. The full study protocol has been approved by the Conjoint Health Research Ethics Board at the University of Calgary and *Sub-Study A* of the protocol has also been approved by Research Ethics Boards at the University of British Columbia; Vancouver Coastal Health Authority Research Institute; Interior Health Authority, British Columbia; and St. Michael’s Hospital.

### Theoretical framework

**Figure 1:**
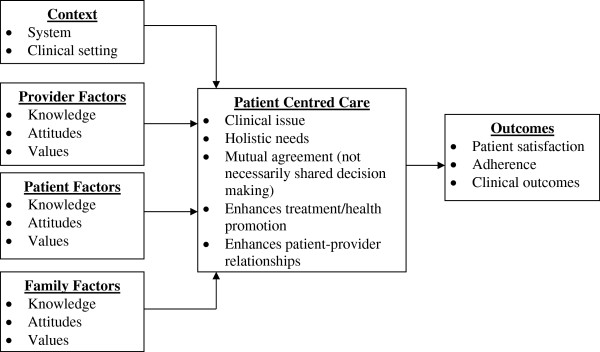
Theoretical framework of patient and family-centred care.

This research will examine the quality of care provided to critically injured patients using a theoretical framework of patient and family-centred care (Figure 1) and a conceptual model of QIs of injury care (Table 1). We developed a theoretical framework of patient and family-centred care that is informed by patient, family, provider and contextual factors and derived from the work of Gillespie et al. [38], Mead et al. [39] and Stewart [40]. Based on our previous work [18], we developed a conceptual model of QIs in injury care that merges the Donabedian framework of health care quality (structure, process, outcome) [41-43] with components of a trauma system (prehospital care, hospital care, posthospital care, secondary injury prevention) [44].

**Table 1:** Conceptual model of quality indicators of trauma care^*†^

**Phase of care**	**Structure**	**Process**	**Outcome**
**Prehospital**	protocol for field triage	time to first medical contact	death
**Hospital**	massive transfusion protocol	massive transfusion protocol activation	adverse event
**Posthospital**	rehabilitation referral protocol	evaluation of functional status	multiple hospital visits
**2° Prevention**	chemical dependence screening protocol	chemical dependence screening	recurrent injury

*Table populated with sample clinical QIs [18,45,46].

†Current proposal focuses on acute care including posthospital transition and initiation of 2° prevention.

*Structure* is the environment in which health care is provided and includes material and health resources, operational factors, and organizational characteristics of the healthcare facility.

*Process* is the method by which healthcare is provided and includes the giving and receiving of care by the providers and healthcare system.

*Outcome* is the consequence of healthcare and includes the health status of patients and communities.

### Sub-study A: patient & family focus groups

#### Design

With little prior research to guide the content and format of patient and family-centred QIs of injury care, qualitative research is needed to develop a comprehensive appreciation of patient and family perspectives. Focus group methodology is an effective technique for exploring attitudes and needs, and the contextual factors that influence those perspectives [47]. The aim of these focus groups is to describe the preferences, needs and values of critically injured patients and their families regarding the quality of health care delivered.

A grounded approach will be used to elicit and understand participant views about the most important dimensions of quality of injury care; how quality of injury care should be measured; how quality should be reported; and how quality should be improved [48]. Rigour will be optimized by sampling from a range of trauma centres featuring varying institutional and patient characteristics that could influence patient experiences, exploring responses inductively for emerging ideas based on a conceptual framework, extending the conceptual framework by thoroughly examining emerging themes including deviant cases, demonstrating responses from an array of respondents by including an anonymous identification code with exemplary quotes, and comparison of independent thematic coding across two individuals [49].

#### Sampling and recruitment

A convenience sample of consecutive consenting patients and family members will be recruited. The research coordinators (RC) will identify critically injured adult (≥18 yrs) patients admitted to each trauma centre over a 12 month period (n ≈ 125-175 patients/centre). Patients will be eligible if they are admitted to hospital for penetrating or blunt polytrauma injury (i.e. no isolated burns or drownings); during admission were admitted to an intensive care unit, a step-down unit or a monitored, high acuity unit; and have an anticipated length of stay of at least seven days. These criteria are designed to identify patients with major injuries who are likely to experience sufficient exposure to health care to be able to provide commentary on quality of injury care. Family members will be eligible to participate if their family member (patient) meets the aforementioned criteria and the family member visited the hospitalized patient, at least one time during their hospitalization. Patients and family members will be eligible if they speak English. Recovering patients and their family members will be approached by the site RC prior to hospital discharge and asked whether they would be willing to be contacted in the future for potential participation in a forthcoming focus group. Contact information will be collected for potential participants. For patients who die during their hospital stay, families will be mailed a bereavement package four weeks after death that includes an invitation to share their experiences and participate in our study. Each site will conduct a focus group of patients and families of survivors and a second group for families of non-survivors. Focus groups will be conducted until thematic saturation is achieved.

#### Data collection

Patients and family members that express interest in participating will be contacted by telephone or email and asked whether they would like to participate in a focus group discussion. Patients/family members that consent to participation will be scheduled for a focus group. Verbal consent will be obtained at the time of telephone screening and written informed consent will be obtained prior to the focus groups. It is expected that the RCs will encounter patients recovering from a severe traumatic brain injury who are incapable of understanding our study procedures. Accordingly, a modified version of the consent-capacity assessment tool will be used to evaluate whether patients are able to understand and consent to study procedures [50]. Once five to eight participants agree to attend a focus group, recruitment for that group will end.

The focus groups will be moderated by a trained facilitator (AG). A clinical co-facilitator with extensive experience in the delivery of injury care (HTS) will be present at each group to address any clinical questions or concerns raised during the group. The moderator will follow a semi-structured focus group guide designed to elicit participants’ experiences with care and their perceptions of what constitutes good quality care (see Additional file 1). Each focus group will include an overview to introduce the purpose and agenda, an icebreaker exercise to familiarize the participants with each other, a series of questions which proceed from general to specific, and a summary to highlight and verify key points [47], including patients’/family members’ knowledge, attitudes and values surrounding patient-centred care and outcomes of care. Participants will be asked to complete a brief written questionnaire prior to the start of the focus group that will collect demographic data including age, sex, marital status, mother tongue, ethnicity, religion, postal code (residential location), education, patient injuries and patient functional status (pre-injury, present).

#### Data analysis

All focus groups interviews will be audio taped, transcribed verbatim, assigned a unique identifier and imported into *MAXQDA2* (Verbi Software, Marburg, Germany) a computer program for qualitative data management. Qualitative content analysis of the transcripts will begin after the first completed focus group and will draw on grounded theory using a process of open, axial and selective coding [51,52]. Two investigators (JB, AG) will independently read each transcript and code the raw data, line by line. Axial coding will be done to examine the context, intervening conditions and consequences of core variables. For example, the investigators will identify what contextual factors influence the identification of exemplary cases of high quality injury care [53]. Selective coding is the final stage of analysis in which a ‘story’ of patient and family perceptions of what constitutes quality injury care is built. Written memos will provide a record of the analytic process [51,52].

### Sub-study B: multi-step quality indicator development process

#### Design

We will develop patient and family-centred QIs of injury care using a rigorous deliberative process, involving a panel of national injury stakeholders. The panel will be presented with a list of themes identified in the *Patient and Family Focus Groups (Sub-Study A)* and a summary of the findings. The themes will be presented in the form of QIs (e.g. provider team should offer to meet with patient and/or family within 24 hours of admission to hospital) [27]. A modified version of the RAND/UCLA Appropriateness Method (RAM), a reproducible and valid nominal group technique used in health services research to gather feedback and information from relevant experts [54-57], will be utilized. The methodology will allow the combination of a highly structured process for rating QIs and an interactive meeting to explore areas of disagreement and review of QI definitions, data elements and codes.

#### Sampling and recruitment

A national panel of stakeholders involved in the multi-disciplinary care (emergency medical systems, subspecialty hospital care, rehabilitation, secondary injury prevention), organization (organizational leadership & healthcare quality) and advocacy (patient/family advocates) of critically injured patients will be assembled by seeking nominations through the Trauma Association of Canada (http://www.traumacanada.org), Ontario Neurotrauma Foundation (http://www.onf.org), Brain Injury Association of Canada (http://www.biac-aclc.ca) and the Canadian Institute for Health Information (http://www.cihi.ca). Nominated members will be selected to obtain broad expert and geographic representation. This group of stakeholders will also be asked to nominate other stakeholders in related disciplines. After establishing a list of potential candidate members for our panel we will approach them with details of the time requirements and program details. Interested nominees will be invited to participate in panel. A target sample size of 9 panel members is based on the RAM and should allow obtaining broad expertise and geographic representation [57].

#### Data collection

A survey consisting of a list of all of the potential QIs proposed or identified from themes in the *Patient and Family Focus Groups (Sub-Study A)* will be developed. Panel members will be mailed a one-page monograph for each QI describing the indicator (definition, specifications for numerator, denominator, etc.), and summarizing the evidence from the *Patient and Family Focus Groups (Sub-Study A)* in the form of direct quotes from focus group participants with descriptions of discussion in the focus groups. Panellists will be asked to independently rate each QI according to four dimensions derived from the Strategic Framework Board in the United States: [58] 1) targets important improvements in the care of critically injured patients, 2) feasible to implement, 3) easy to use, 4) strength of scientific evidence (using the GRADE criteria) [59]. These dimensions will be ranked on the validated nine -point RAM scale with one representing strong disagreement and nine representing strong agreement [57]. Overall assessment of the QI will be scored on a nine-point scale as unnecessary (1–3), supplementary (4–6) and necessary (7–9) [57]. The median rating will be used to classify each item. Disagreement on the ratings for a QI will be defined as an overall assessment by at least three panellists (one third) in the unnecessary range and at least three ratings in the necessary range [57]. Panellists will be asked to provide written comments, and to suggest additional QIs.

Potential QIs with a median overall assessment score of one to three (unnecessary) will be removed. All other QIs including newly suggested indicators will be retained for the second round questionnaire. A second round questionnaire will be prepared and mailed to all the panellists with a frequency distribution breakdown of scoring for all potential QIs from round one. Panellists will be asked to score the QIs including the newly suggested indicators using the same scales as in round one and comments will be solicited. Once all of the second round questionnaires have been received and scored, they will be collated using the same methods described for round one.

A two-day workshop will be held with the stakeholder panel to review the retained QIs. Each QI will be discussed and independently re-scored using the same scale as in round one. Indicators with a median overall assessment score of seven or greater (necessary) will be retained. All QIs with panel disagreement (previously defined) will be rejected. For each retained QI, based on available evidence (*Patient and Family Focus Groups (Sub-Study A)*) and their expertise, panellists will be asked to determine content validity, establish clear definitions of all terms and specify modifications [60]. Deliberations will be performed as a group and final agreement on QI specifications will be established using the RAM scales.

#### Data analysis

Standard definitions and a data dictionary for the QIs retained from round two will be developed before the panel members meet face-to-face [61]. In addition, QIs dependent on trauma registries or administrative data will have coding algorithms developed [62]. Two expert coders experienced in International Statistical Classification of Diseases and Related Health Problems, 10th Revision (ICD-10), coding will independently code the QIs using the ICD-10 computerized ‘code finder’. The two lists of codes for each QI will then be combined. After excluding duplicate codes, a comprehensive list of codes will be developed for each QI. The codes will be described using clinical terms in the ICD-10 manuals. Following the workshop, the coding algorithms for the retained QIs will be jointly revised by the two expert coders to both accommodate the panel’s recommended modifications and ensure consistency with the clinical definitions established.

### Sub-study C: pilot test of quality indicators

#### Design

The findings from the *Patient and Family Focus Groups (Sub-Study A)* and the *Multi-Step Quality Indicator Development Process (Sub-Study B)* will inform the specific analyses performed in the *Pilot Test of Quality Indicators (Sub-Study C)*. First, the QIs will be implemented in the form of a telephone survey of patients and family members following hospital discharge at the four trauma centres that participated in the *Patient and Family Focus Groups (Sub-Study A)*. Second, the measurement properties for each QI will be evaluated, including construct validity and reliability. Third, a clinical sensibility assessment of the QIs will be performed [63].

#### Sampling and recruitment

The same method for identifying and approaching potential participants used in the *Patient and Family Focus Groups (Sub-Study A)* will be followed to recruit patients and family members for *Pilot Test of Quality Indicators (Sub-Study C*).

#### Data collection

The same sequential approach for data collection used in the *Patient and Family Focus Groups (Sub-Study A)* will be followed. Patients and family members will be contacted by the site RC following hospital discharge (4 weeks for patients discharged home, 12 weeks for patients discharged to rehabilitation or deceased) and offered an opportunity to participate in a four part telephone survey evaluating their recent injury care experience: 1) patient and family-centred QI survey, 2) patient and family satisfaction survey, 3) clinical sensibility survey and 4) demographic and clinical data survey. The RC will contact a randomly selected 25% of participants who completed the survey instruments a second time, one week following initial survey administration and re-administer the patient and family-centred QI survey to evaluate intra-rater reliability.

1) Patient and family-centred quality indicator survey: a survey instrument will be developed to administer the QIs developed in The *Multi-Step Quality Indicator Development Process (Sub-Study B)*. Two versions of the instrument will be developed; one for surviving patients and their family members and a second for family members of non-surviving patients. Except for pronouns (e.g. “his or her injuries” instead of “your injury”), the family member survey (survivors) will be identical to the patient survey. The indicators will be designed to report patient and family observations and will be structured as binary responses. Family members will be instructed to report their own views and not to offer proxy responses on behalf of the patient.

2) Patient and family satisfaction survey: The Patient Satisfaction with Injury Care Survey [64] (43 acute care items and 27 post-acute care items) will be administered to measure patient and family satisfaction with injury care.

3) Clinical sensibility survey: A clinical sensibility assessment will be performed of the QIs based on Feinstein’s criteria by asking patients and family members to rate each QI for clarity, utility, face validity, content validity, redundancy, discriminability (distinguish between patients and family members receiving good care and poor care) and overall level of importance (see Additional file 2) [63]. Patients and families will be invited to provide suggestions on how to make the QIs better and improve the quality of injury care.

4) Demographic and clinical data: The RC will collect basic demographic and clinical data from patients and family members including age, sex, marital status, ethnicity, religion, education, patient injuries and patient functional status (pre-injury, present). The RC will request permission from patients to obtain injury data from the trauma registries.

#### Data analysis

The goals of the *Pilot Test of Quality Indicators (Sub-Study C)* is to evaluate the feasibility of implementation, construct validity, reliability and clinical sensibility of the patient and family-centred QIs developed in the *Multi-Step Quality Indicator Development Process (Sub-Study B)*. Global data will be presented on the proportion of patients and families who report receiving care that satisfied each QI along with binomial 95% confidence intervals. Detailed tabulations will be presented by respondent characteristics (e.g. type of injury, whether a patient or family member etc.).

Construct validity will be defined as the extent to which the QIs relate to other measures in a manner consistent with a theoretically-derived hypothesis concerning the domains measured [65]. Validity will be determined using two approaches. First, the agreement between the individual QIs (binary measures) and overall quality of care scores (sum of the QIs) will be examined at the patient level. Second, the agreement between QIs (both individual QIs and overall quality of care scores) and patient and family satisfaction survey ratings (ordinal scale) will be examined. Spearman rank-correlation coefficients and Wilcoxon Rank sum tests will be calculated for agreement between QIs and overall patient and family satisfaction and individual domains of the satisfaction survey. Reporting of health care quality and evaluations of satisfaction with care are not the same, but do measure similar domains of care and provide a means for evaluating construct validity [23,66]. Kappa values will be calculated to evaluate intra-rater reliability for individual QIs while intraclass correlation coefficients will be calculated to evaluate overall quality of care scores. Clinical sensibility measures (six measures) and overall level of importance will be summarized for each QI as medians with interquartile ranges.

A sample size of 72 participants for intra-rater reliability is sufficient to detect a true kappa of 0.85 in a one-sided test for a kappa less than or equal to 0.65 under the null hypothesis with 80% power and a significance level of 0.05 [67]. Therefore, for a re-sampling rate of 25% to assess intra-rater reliability, a total sample size of 360 telephone survey participants is needed (accounting for 20% loss to follow up). A sample of this size will also provide adequate power to detect a true intraclass correlation of 0.85 for a one-sided test for an intraclass correlation less than or equal to 0.65 under the null hypothesis using an F-test with a significance level of 0.05 [68]. Statistical analyses will be performed using SAS version 9.2 (SAS Institute Inc., Cary, NC).

## Discussion

Measuring the quality of injury care is but a first step towards improving patient outcomes. The above-described approach to gathering data from patients/families is feasible and likely to succeed. Members of our research team have previously conducted focus groups of decision support tools [69,70] and development of QIs [71,72].

This research is essential because it will develop the first set of patient and family-centred QIs of injury care. Around the world, countries are faced with a quietly growing injury epidemic [2]. Yet remarkably, little is known about the quality of injury care and the impact on outcomes that patients value. To improve patient care, we need accessible, reliable indicators of quality that are important to patients, and that can then be used to establish quality of care benchmarks, to flag potential problems or successes, follow trends over time and identify disparities across organizations, communities, populations and regions. The proposed work builds directly on our existing research program of employing clinical research evidence to measure the quality of injury care by incorporating patient and family preferences, needs and values into the development of an applied health tool that will allow health care providers to develop local quality improvement initiatives, systems managers to identify and correct system wide problems, policy makers to plan for future trauma systems and funding agencies to establish priorities for future injury research.

## Abbreviations

CAHPS: Consumer assessment of health providers and systems survey; HCAHPS: Hospital version of consumer assessment of health providers and systems survey; ICD-10: International statistical classification of diseases and related health problems, 10th revision; IOM: Institute of medicine; QI: Quality indicator; RAM: RAND/UCLA appropriateness Method; RC: Research coordinator.

## Competing interests

The authors declare that they have no competing interests.

## Authors’ contributions

HTS was involved in the conception of the study and participated in obtaining funding, designing the study and the drafting and editing of the manuscript. JB participated in the design of the study, its coordination and the editing of the manuscript. SS was involved in the conception of the study and participated in obtaining funding, designing the study and editing the manuscript. AG was involved in the conception of the study and participated in obtaining funding, designing the study and editing the manuscript. All authors have read and approved the final manuscript.

## Pre-publication history

The pre-publication history for this paper can be accessed here:

http://www.biomedcentral.com/1472-6963/13/31/prepub

## Supplementary Material

Additional file 1 **Draft Focus Group Guide. **Description of data: draft of the semi-structure guide for the patient and family focus groups in *Sub-Study A.* Click here for file

Additional file 2 **Draft Clinical Sensibility Instrument. **Description of data: draft of survey instrument to measure clinical sensibility in *Sub-Study C.* Click here for file
