# Supplementary material for: Developing a patient and family-centred approach for measuring the quality of injury care: a study protocol
Source: BMC Health Serv Res. 2013 Jan 27;13:31. doi: 10.1186/1472-6963-13-31 (PMC3570378; doi:10.1186/1472-6963-13-31)
Supplement: Additional file 1 — Draft Focus Group Guide. Description of data: draft of the semi-structure guide for the patient and family focus groups in Sub-Study A. [file 1472-6963-13-31-S1.pdf]

Additional file 1. Trauma Quality of Care Focus Group Discussion Guide

INTRODUCTIONS and OVERVIEW (20 min)

- You, me (name, position, affiliations, professional roles and responsibilities and interests)
- Each participant (name, where from)
- Explain the purpose of the project (Tom) and thank them for participating
- Explain the purpose of the focus group, what we will do, how the information will be used (Anna)
  - Health services research different from lab research, focus is on improving care
  - Some researchers use quantitative methods. That means they analyze and summarize data/numbers about how many people get sick or injured, and how many received a particular treatment, and how many got better but that doesn't provide us with information about how to improve health care services.
  - Researchers like me use qualitative methods. That means we talk to people including health professionals, managers, government policy makers, and patients and families to learn more about how care is delivered and how it can be improved
  - So Tom invited me to speak with you to discuss your recent injury experiences
  - We will ask you to identify elements that were satisfying and less satisfying. You may not want to reveal the exact nature of the injury and that's ok. Primarily we are interested in identifying patient and family expectations, and we are asking you to reflect on your injury experience only so that you can explain what about injury care is important and meaningful
  - Ultimately we want to produce a list of criteria that you and other patients and families feel should be essential components of injury care, and that list may become a standard used across Canada and elsewhere by which health care facilities plan the services they deliver, and they or other organizations like governments evaluate the quality of the services that are delivered
  - So we are asking you to reflect on your experiences to suggest those criteria or standards, called quality indicators, that you and other patients and families believe should always be available, and which may become standards by which health care professionals and researchers evaluate and report on the actually quality of care
  - For each topic we discuss, we will give each of you an opportunity to express an opinion, and will then open it up for general discussion. After discussion of each topic Tom and I will summarize the key criteria or indicators suggested by your opinions and discussion to confirm that we understood correctly

CLINICAL CONTEXT/PERCEPTIONS (20 min)

Think back to your experience of injury care and in a few words name what about it was particularly good or not good. Let's start with good.

<u>Prompts:</u>

What made it good/bad?

Was it the same/better/worse than other health care experiences you have had? Why or how?

What key expectations/concerns did you have during the experience? How were they addressed?

Ask them to consider pre-hospital, hospital, and post-hospital care

Ask the them to consider processes of care, functional outcomes, other outcomes

RECAP: note suggested indicators

MUTUAL AGREEMENT / PATIENT-PROVIDER RELATIONSHIP (15 min)

How satisfied were you with the medical team that cared for you (doctors, nurse, therapists, others)?

What about them or what they did resulted in your impression?

Did you have as much opportunity as you wanted to be informed, ask questions, be involved with decisions?

What would you have wanted to be different?

RECAP: note suggested indicators

HOLISTIC NEEDS / SUPPORTIVE CARE (15 min)

Apart from clinical needs or information needs directly related to the injury, what other needs did you or your family experience?

In what way were they addressed?

What would you have wanted to be different?

RECAP: note suggested indicators

PATIENT REPORTED OUTCOMES (20 min)

As we noted earlier, one of our goals is to develop a list of criteria or standards that we and others can use to measure the quality of injury care; in that way we identify problems and can work toward improving them.

- Review the criteria/indicators
- Ask for others not already prompted for or mentioned
- Ask the group to identify priorities and reasons for the priorities (depending on how many were generated overall and the time, we could ask them to informally achieve consensus on the top three or five or ten)
- Ask them which should be measured and publicly reported, versus confidentially communicated to governments and hospitals and health care professionals

QUESTIONS / CONCLUSION (5 min)

Ask if there are any questions about the overall research project, the focus group, what happens next?

Thank them for participating?

Will we send them a summary of the findings? If so we need to get their contact info.
