# Supplementary material for: Developing a patient and family-centred approach for measuring the quality of injury care: a study protocol
Source: BMC Health Serv Res. 2013 Jan 27;13:31. doi: 10.1186/1472-6963-13-31 (PMC3570378; doi:10.1186/1472-6963-13-31)
Supplement: Additional file 2 — Draft Clinical Sensibility Instrument. Description of data: draft of survey instrument to measure clinical sensibility in Sub-Study C. [file 1472-6963-13-31-S2.pdf]

Additional file 2. Draft Clinical Sensibility Instrument

How are we doing?

Your Opinions about Our Patient and Family-Centred Quality Indicator Survey

1. Please rate the patient and family-centred quality indicators using the following criteria:

	**Agree** 1	2	3	4	**Disagree** 5
Simple and easy to understand	1	2	3	4	5
Likely to elicit honest information about quality of injury care	1	2	3	4	5
Directed at important elements of quality of injury care	1	2	3	4	5
Missing important patient and/or family perspectives on quality of injury care	1	2	3	4	5
Includes duplicate items	1	2	3	4	5
Likely to distinguish between patients and families receiving good care and poor care	1	2	3	4	5
An important tool for measuring, reporting and improving the quality of injury care	1	2	3	4	5

2. What suggestions do you have to improve the patient and family-centred injury quality indicators?

____

____

____

____

3. What suggestions do you have to improve the quality of injury care?

____

____

____

____
